# Experimental Verification of the Concept of Using LOFAR Radio-Telescopes as Receivers in Passive Radiolocation Systems †

**DOI:** 10.3390/s21062043

**Published:** 2021-03-14

**Authors:** Julia Kłos, Konrad Jędrzejewski, Aleksander Droszcz, Krzysztof Kulpa, Mariusz Pożoga, Jacek Misiurewicz

**Affiliations:** 1 Institute of Electronic Systems, Faculty of Electronics and Information Technology, Warsaw University of Technology, Nowowiejska 15/19, 00-665 Warsaw, Poland; julia.klos.stud@pw.edu.pl (J.K.); aleksander.droszcz.stud@pw.edu.pl (A.D.); k.kulpa@elka.pw.edu.pl (K.K.); jmisiure@elka.pw.edu.pl (J.M.); 2 Space Research Centre Polish Academy of Science, Bartycka 18A, 00-716 Warsaw, Poland; pozoga@cbk.waw.pl

**Keywords:** passive coherent localization, passive radar, radiolocation, bistatic radar, radio-telescope, LOFAR, space surveillance, space object tracking

## Abstract

The paper presents a new idea of using a low-frequency radio-telescope belonging to the LOFAR network as a receiver in a passive radar system. The structure of a LOFAR radio-telescope station is described in the context of applying this radio-telescope for detection of aerial (airplanes) and space (satellite) targets. The theoretical considerations and description of the proposed signal processing schema for the passive radar based on a LOFAR radio-telescope are outlined in the paper. The results of initial experiments verifying the concept of a LOFAR station use as a receiver and a commercial digital radio broadcasting (DAB) transmitters as illuminators of opportunity for aerial object detection are presented.

## 1. Introduction

Passive coherent location (PCL) radars are a special type of radar that detect and track objects without emitting own waveform [1,2,3,4,5]. Instead of possessing a cooperative transmitter they exploit different existing transmitters as illuminators of opportunity. The most popular illuminators of opportunity employed in passive radiolocation systems are analog and digital radio transmitters, digital television transmitters or even cellular base stations. The lack of own dedicated illuminator makes a passive radar undetectable and relatively inexpensive. The development of different telecommunication and broadcast networks with increasing number of transmitters has a strong effect on the popularity of passive radiolocation. A passive radar directly measures bistatic parameters of the target, i.e., a bistatic range and a bistatic velocity. Other target parameters such as position, velocity and effective radar cross section might be also estimated, using multiple illuminators of opportunity and/or multiple receivers.

LOFAR (LOw-Frequency ARray for radio astronomy) is an international network of radio-telescopes designed and constructed by ASTRON, the Netherlands Institute for Radio Astronomy. It started operating in 2012. Presently, LOFAR consists of 52 independent LOFAR stations, including 38 stations operated by ASTRON and 14 international stations located throughout Europe. Three of these stations are in Poland, near Poznań, Olsztyn and Kraków. Since a LOFAR station is a large sky-looking antenna array which can receive radio signals within the bands used by typical broadcasting transmitters, it is a good candidate for a passive radar receiver.

The purpose of this paper is to verify whether it is possible to use the signals received by a LOFAR station to detect aerial or space targets without any additional modification in a LOFAR station itself. Our goal is to achieve, in the near future, the ability to detect and track satellites and space debris using the existing LOFAR hardware infrastructure and relatively cheap passive radar technology, adding only adequate signal processing capabilities. The motivation for our research was that we obtained more than 300 km detection range of aerial targets using simple low-gain antennas as receivers, as well as the reports from papers presented by other authors describing their work on passive detection of meteors and space objects [6,7].

While LOFAR primarily explores the edge of the Universe and solves the mysteries of astronomy, it can also be used to observe at shorter distances, for example airplanes using passive radar technology. Furthermore, due to the size of LOFAR antenna and the number of LOFAR stations located in almost whole Europe, it is likely to observe the satellites using the network of LOFAR stations and the multiple illuminators of opportunity, located in different countries within Europe. The experiments presented in the paper show the detection of air targets using one LOFAR station with digital radio (DAB) transmitters of opportunity. The success of these experiments serve as a proof of concept, allowing the future extension towards detecting and tracking satellites with the same technique. This paper is an extension of a paper originally presented in 2020 at the 21st International Radar Symposium [8]. Another paper related to particularities of beamforming of a LOFAR station for passive radiolocation purposes [9] was also presented during the 21st International Radar Symposium in 2020.

## 2. LOFAR Based Passive Radiolocation

### 2.1. LOFAR Network and a LOFAR Station Structure

**Figure 1:**
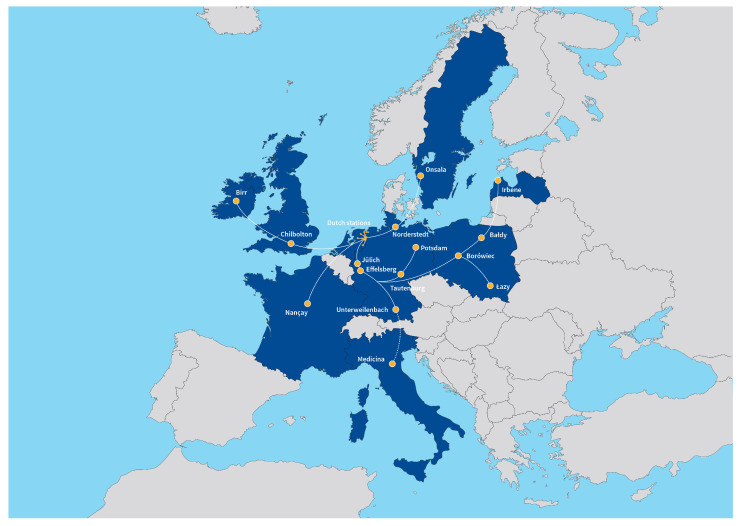
Existing and planned LOFAR stations. Credit: ASTRON.

The LOFAR network of radio-telescope stations consists of 38 stations located in the Netherlands, operated directly by ASTRON, and 14 international stations located in Germany (6), Poland (3), France (1), Sweden (1), the United Kingdom (1), Ireland (1) and Latvia (1). Figure 1 shows the location of the LOFAR stations in Europe. The LOFAR stations working together form the largest radio-telescope operating at the lowest frequencies for which the space observation can be carried out from the Earth [10].

**Figure 2:**
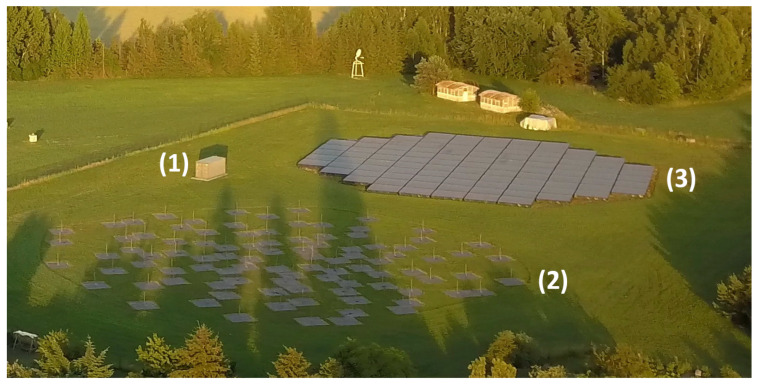
LOFAR PL610 station in Borowiec, Poland, owned by SRC PAS. From left to right: (1) container with electronic equipment, (2) LBA antennas, (3) HBA antennas).

Each LOFAR station consists of two fields of distinct antenna types: Low-frequency Band Antennas (LBA) operating in a frequency range between 10 and 90 MHz, and High-frequency Band Antennas (HBA) operating in the frequency range between 110 and 250 MHz [11]. All LOFAR stations are connected by fiber optic links to the central LOFAR processor in Groningen, the Netherlands. Each station can work in two ways: in the International LOFAR Telescope (ILT) mode—when the signals and data from each station are sent to the central processor in Groningen, and in the stand-alone mode when the signal and data processing is controlled by the station owner. Figure 2 shows the LOFAR PL610 station in Borowiec, Poland, owned by the Space Research Centre, Polish Academy of Science. The experiments whose results are presented in this paper were performed using the HBA antennas in this station.

**Figure 3:**
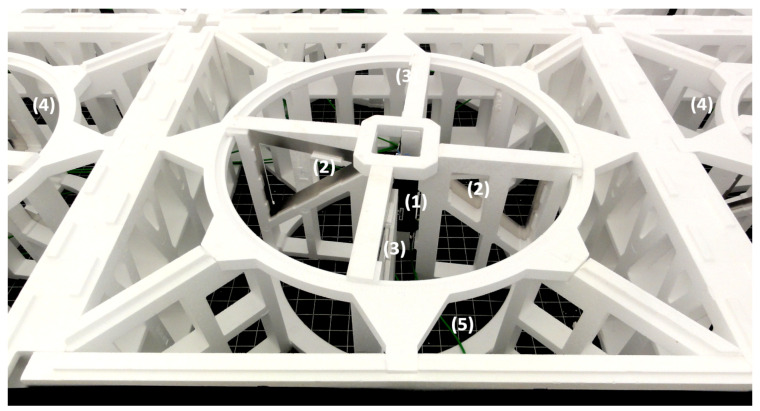
HBA tile internal structure. 1—preamplifier, 2—dipoles for one polarization, 3—dipoles for second polarization, 4—adjacent dipoles, 5—connection to amplifier and combiner.

The HBA field consists of 1536 pairs of antennas grouped in 96 tiles. Figure 3 shows the internal structure of a single antenna. The antenna consists of two horizontal dipoles oriented perpendicularly to receive two linear polarizations. The dipoles in a single tile are arranged in a 4 by 4 square array containing 16 pairs of antennas closed in a 5 × 5 m container (Figure 2). The tiles are arranged next to each other, forming a structure resembling a circle with a diameter of approximately 62 m. Within a single tile, the signals can be shifted in time by 0–65 ns with a 5 ns step and summed to form a directed beam [11]. Such a process is called the analog beamforming. Each dipole is equipped with a low-noise preamplifier to improve the signal-to-noise ratio (SNR) and a programmable attenuator (0–7.25 dB with a 0.25 dB step). The HBA tile receives signals in a range of 110–250 MHz and the proper frequency range for analog-to-digital converters (ADC) is selected in the receiver by the use of switchable filters [12]. Signals from the HBA tiles are sampled separately by 192 ADCs with a sampling frequency of 200 MHz and with 12 bit resolution—96 ADCs for each polarization. Further processing of the signals is digital.

Typically, during the standard use of a LOFAR station by astronomers, the station shifts digitally signal phases from selected tiles and combines the signals to form a beam in a selected direction. The output of this procedure is divided into subbands of 195.3125 kHz each and sent in real time to a data logger. This mode allows recording the signals directly for the required band and the defined set of tiles. What is more, this mode allows simultaneous creation of independent beams in defined directions. The limit is the signal bandwidth of 50 MHz in 16 bit mode and 100 MHz in 8 bit mode. Thus, for the DAB+ signal (2 MHz bandwidth for a single channel) we can form over 20 simultaneous beams with 16-bit resolution.

The LOFAR station hardware enable also the acquisition of short sequences (maximally of the length of 1.4 s) of raw signals with the sampling frequency of 200 MHz, directly from all 192 ADCs using the transient buffer boards (TBB) [10,11,12]. The TBBs allow the processing of the signals from all tiles of a LOFAR station without any data processing performed in a LOFAR station. However, the transmission time of such an amount of data to the data logger is about 15 min. Nevertheless, in the initial phase of our research on the use of a LOFAR station as a receiver in the passive radiolocation system, we decided to use this mode which enables the access to the raw signals registered by LOFAR. The TBB module was originally designed for the observation of cosmic ray air showers emitting short radio pulses and other short-term phenomena called transit in the LOFAR nomenclature. For example it is currently used to observe time evolution of lightning discharges, where it is required to record signals in very short periods of time at the moments of the occurrence of the phenomenon. The start of recording is synchronized by the dedicated signal from a particle detector or by a software request, as in our research, which translates into a small amount of transmitted data, therefore the speed of data transmission was not a priority. The TBBs are also used for internal diagnostics of LOFAR stations.

### 2.2. Passive Radar Concept

The active radar emits probing (sounding) pulses and listens for target echoes. The time delay between sending the sounding pulse and reception of the target echo is proportional to the target distance and the Doppler shift between the probing pulse and the echo carries information about the target radial velocity. The spatial (angular) information of the target position is obtained by scanning by the radar beam and finding the coordinates of the maximum of the target echo power. The alternative approach is to divide the antenna array into sub-arrays and analyse the phase differences of the echo signal received by each of sub-apertures.

The passive radar does not emit any signal but instead it is tuned to the selected high-power transmitter of opportunity. It operates by receiving and processing two signals—a direct signal from the transmitter and an indirect signal reflected from the target of interest. As most commonly selected transmitters are transmitters of analogue or digital radio (DAB—Digital Audio Broadcasting) or digital television (DVB—Digital Video Broadcasting), they are omnidirectional at least in azimuth plane and illuminate all the surveillance space [13]. The vertical characteristics of such transmitters are usually not omnidirectional, as most of the subscribers are located on earth surface. Thus, the beam is rather narrow in the vertical dimension: 3–15°. To detect the high altitude targets it is necessary to use a distant transmitter for which the main transmitting beam can illuminate the target.The detection range of the passive radar is predicted by formula:

(1) $$R_{max}=\sqrt[{4}]{\frac{P_{t}G_{t}G_{r}S_{0}\lambda t_{i}}{{\left(4\pi \right)}^{3}LD_{0}kT_{r}}},$$

where $P_{t}$ is the Effective Isotropic Radiated Power (EIRP) of illuminator, $G_{t}$ is the transmitter antenna gain towards target, $G_{r}$ is the receiver antenna gain towards target, $S_{0}$ is the target radar cross-section, λ is the illuminating waveform wavelength, *L* stands for system losses including Noise Factor, $D_{0}$ is the detection threshold, *k* stands for the Boltzmann constant $1.38064852\times 10^{-23}$ m^2^ kg s^−2^ K^−1^, and $T_{r}$ is the receiver temperature (effective). The passive radar designers can influence only on one parameter—the integration time. For a classical passive radar the integration time between 0.1 to 1 s is usually assumed, as with such an integration time a target usually stays in a single range and Doppler resolution cell. With longer integration times it is necessary to compensate both range and velocity walk using stretch [14,15,16,17,18,19,20] and acceleration [21] processing.

The maximum detection range (1), valid for the monostatic case, is frequently used to show the detection potential of the passive radar– then it is called a quasi-monostatic detection range. In the case of the active monostatic radar the target-transmitter and target-receiver distances are equal. In the multistatic passive radar they are usually different and the range $R_{max}$ expressed by (1) is the geometric mean of these two distances:

(2) $$R_{max}=\sqrt{R_{t}R_{r}}.$$

**Figure 4:**
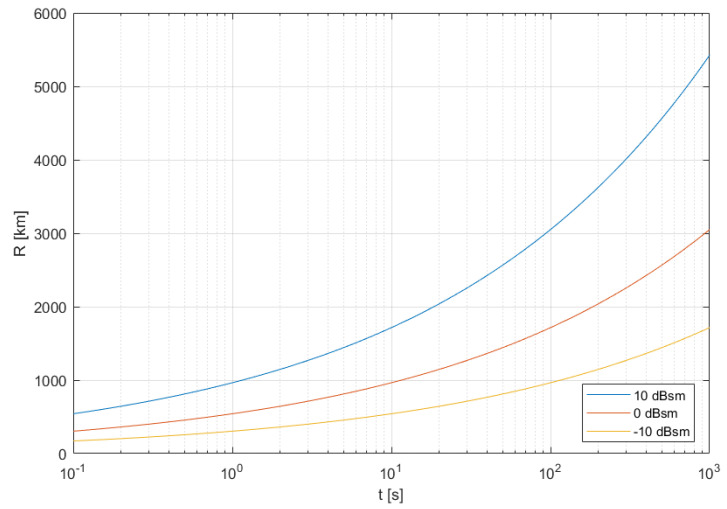
Detection range (quasi-monostatic) for different integration times.

As a result, if a target is close to either receiver or transmitter, the other distance could be much longer. The achievable quasi-monostatic detection ranges for the LOFAR receiver with gain equal to 35 dB and DAB illuminator of power 10 kW (EIRP) with 0 dB gain antenna, assuming 10 dB detection threshold and 10 dB system losses, for three sizes of targets (RCS) 10 dBsm (10 m^2^), 0 dBsm (1 m^2^) and −10 dBsm (0.1 m^2^), are presented in Figure 4. As one can see from Figure 4, for a 1 s integration time it would be possible to detect large space targets (10 dBsm) with orbits up to 1000 km. For a 100 s integration time it would be possible to detect also the small space targets (−10 dBsm) at the same orbit, while the medium space targets (0 dBsm) should be detected with orbits up to 1800 km, and large targets (10 dBsm) with orbits about 3000 km. Of course, for such a long integration time the appropriate motion model and the motion compensation methods have to be applied to avoid mismatch losses during the correlation. It is also necessary to exploit distant transmitters located 1000–3000 km from receiver, which are able to illuminate the high orbits.

**Figure 5:**
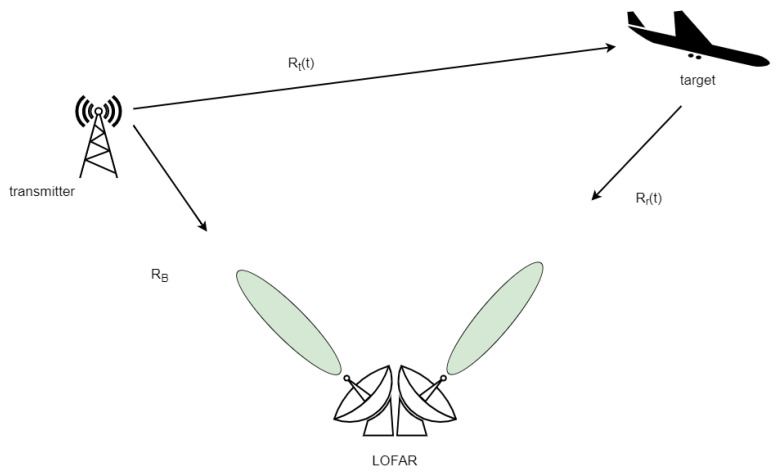
Passive radiolocation setup.

Due to using third-party transmitters that are located far from the receiver, (see Figure 5), the “traditional” monostatic geometry cannot be applied [22]. The bistatic radar measures the time difference between the direct path signal and the reflected target echo arrival. This measurement is related to the bistatic range to a target *R* defined as a sum of transmitter-target distance $R_{t}$ and target-receiver distance $R_{r}$ minus a baseline distance $R_{B}$ (the transmitter-receiver distance) as depicted in Figure 5. The bistatic range equation has the following form (we note that some distances change in time):

(3) $$R\left(t\right)=R_{t}\left(t\right)+R_{r}\left(t\right)-R_{B}.$$

Additionally, the passive radar can also measure the bistatic Doppler shift of an echo, the so-called bistatic velocity defined as a time derivative of the bistatic range. As a consequence of the geometric setup, the bistatic velocity depends not only on the physical velocity vector of an object but also on its position [5].

Target detection is possible if the passive radar is constructed with at least two receiving channels. Compared to conventional radar systems, the passive radar has no information about the transmitted signal as it profits from the illuminators of opportunity. As a result, a dedicated channel for reception of reference signal is required (see Figure 5). The other channels are used to receive the echoes reflected from the observed targets.

Since the antenna of a passive radar does not rotate, so a target is not illuminated periodically, and localising it using the methodology of the classical radar is impossible. With spatial separation of a transmitter and a receiver it is achievable to determine the unambiguous location of a target when multiple pairs of transmitters and receivers are used. Based on the delay between the time of arrival of the surveillance and reference signals a bistatic range is calculated. The measurement of the bistatic range from every pair transmitter-receiver results in a bistatic ellipsoid on which a target can be located. A set of points of the constant bistatic range creates the bistatic ellipsoid with both the receiver and transmitter as the ellipsoid’s foci. Calculating the point of the intersection of multiple ellipsoids provides the estimation of the target position.

**Figure 6:**
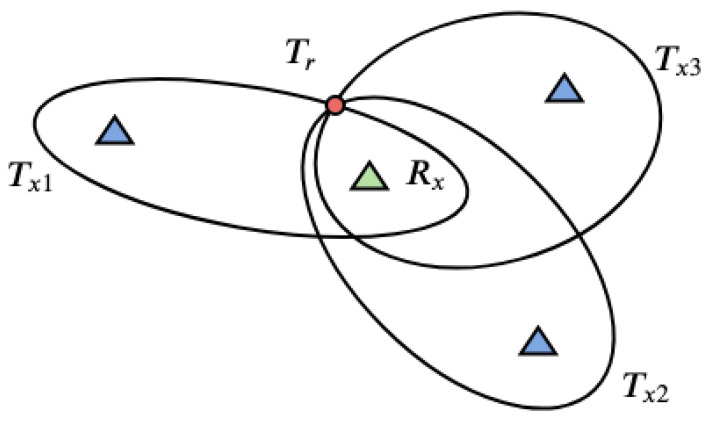
A concept of target localisation as the intersection of ellipses determined by bistatic distances for three different transmitter-receiver pairs.

A minimum of three transmitter-receiver pairs is necessary for three-dimensional location of a target, however resolving the ambiguity may require a fourth pair. Additional pairs improve, in principle, the accuracy and allow the solution of multi-target scenarios. A simplified two-dimensional drawing (for ellipses instead of ellipsoids) illustrating the problem of determining the target position is presented in Figure 6.

### 2.3. Signal Processing for Passive Radiolocation by Means of LOFAR Station

**Figure 7:**
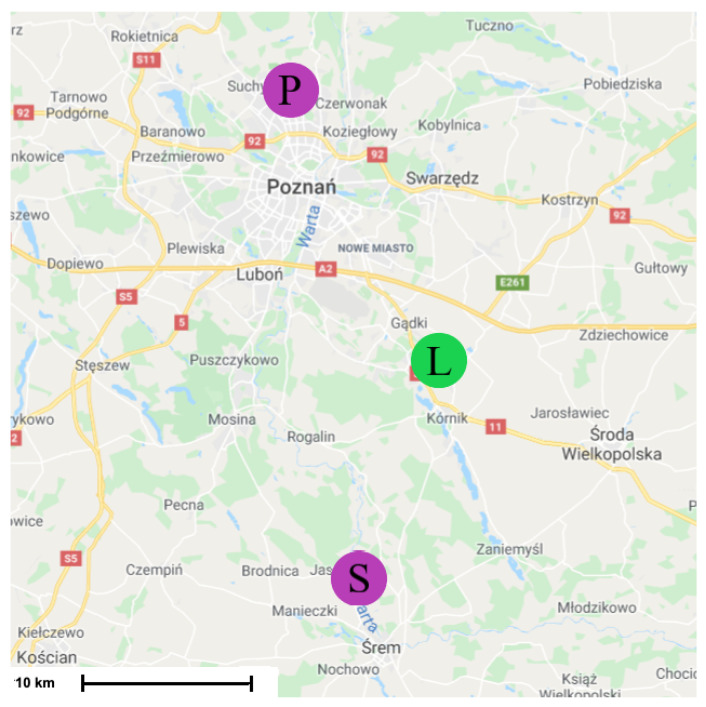
Location of LOFAR station in Borowiec (L) and transmitters in Srem (S) and Piatkowo (P).

The passive radar requires finding external transmitters (illuminators) which illuminate targets of interest, working in the frequency range covered by the LOFAR receiver. In our experiments aimed at verifying the possibility of building a passive radiolocation system employing the LOFAR network, the LOFAR PL610 station in Borowiec was employed together with two digital radio (DAB+) transmitters in Srem and Piatkowo located near this station. A map showing the location of the LOFAR station in Borowiec and the transmitters in Srem and Piatkowo is presented in Figure 7. Both transmitters operate in a single frequency network (SFN) [23] mode with the carrier frequency 223.936 MHz, thus within the frequency band of the LOFAR HBA’s range (110‒250 MHz).

The choice of transmitters was made with a purpose to enable the reception of a direct signal with a reference beam formed using selected tiles of the LOFAR station in Borowiec, as well as to enable the observation of aerial targets. In the case of DAB or DVB-T transmitters, their characteristics have relatively narrow elevation beams directed almost parallel to the Earth’s surface with the beam width from a few to a dozen degrees. Therefore, for the detection of aerial targets such as airplanes the transmitters close to a LOFAR station should be selected. Then, the transmitters illuminate the low-altitude zone, where the aerial objects may appear. For the detection of space objects, much longer baseline distances are needed and distant or very distant transmitters should be used. However, for our experiment with airplanes as targets, the proposed setup alleviated the synchronisation problems that would arise when using distant transmitters, which in turn requires deployment of dedicated reference receivers. The general problem of selecting appropriate transmitters is complex, as it has to address the ambiguity resolution [24] and the location accuracy [25], as well as the problem specific to the observation of orbital targets with a passive illuminator—reaching the orbit of interest with a transmitter beam.

To be able to receive signals directly from the transmitters, the beams of some selected tiles were directed (using analog beamforming) towards the transmitters. Other tiles were steered at the surveillance space (above Swarzedz in Figure 7). The aim of the beamforming was to create the reference and surveillance beams [26]. Summing the signals from relevant channels is perceived as steering beam in the direction of interest. Even after the beamforming, the target surveillance channel still contains significant amount of clutter and direct-path signals, originating from the leakage of the direct-path signal via sidelobes of the surveillance beam, which have usually much higher power than reflected echoes.

**Figure 8:**
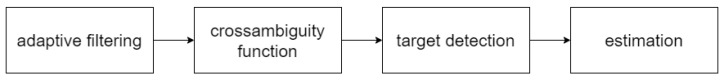
Signal processing stages used in passive radiolocation system.

After the initial preprocessing of received signals including sampling and decimation (in our implementation the decimation rate was 100—from 200 MHz to 2 MHz), the signal processing operations typical for the passive radar were performed, i.e., adaptive filtering, calculation of the cross-ambiguity function (CAF), target detection, estimation of target parameters [5,27] and tracking [6,28,29,30,31]. The sequence of the implemented operations is shown in Figure 8. The most computationally costly procedure is the calculation of the modified CAF with range and Doppler migration compensation which can be perform using different approach such as [7,32,33,34], but computational efficiency and implementations aspects are beyond consideration in this paper.

As was stated before, one of the main problems in the passive radiolocation is the leakage of the reference signal to the surveillance channel, known as a direct-path inference. The power of the reference component in the surveillance channel is usually much higher than power of echoes reflected from targets, which makes the target-related correlation peaks in the CAF invisible. The high-power interference signals are usually cancelled from the surveillance channel by means of adaptive filtering [5,35,36,37]. Removing the direct-path transmitter signal, as well as a clutter and reflections from strong close objects may eliminate the blindness to some targets. Therefore, adaptive algorithms which estimate the impulse response of the illuminator’s channel and follow its changes are commonly used. The copy of the transmitted signal is filtered with the adaptive filter and the output signal is subtracted from the target channel waveform. Several algorithms can be employed to filter the direct-path interference and clutter such as the least mean squares (LMS) algorithm, the recursive least squares (RLS) algorithm, the least squares lattice (LSL) algorithm or the block lattice filter (BLF) algorithm. In the implementation whose results are presented in the paper, the BLF algorithm described in [5] (p. 190) was used. Additionally, to remove the Doppler-spread clutter, we employed the modified block lattice filters, i.e., the BLF algorithm with different frequency shifts around the zero Doppler frequency [5] (p. 192). The exemplary results of such a filtering are clearly visible in Section 3 in Figures 10 and 11.

Next, the key step of radar processing—the CAF calculation—is performed. Mathematically, the CAF is defined as follows:

(4) $$\psi \left(R,V\right)=\int_{0}^{t_{int}}x_{ref}\left(t-\frac{R}{c}\right)x_{meas}^{*}\left(t\right)\exp\left(-j2\pi \frac{V}{\lambda }t\right)dt.$$

and it may be understood as the cross-correlation between the surveillance signal and Doppler shifted versions of the reference signal [38]. Since most of received signal is noise, the correlation peak occurs when the pattern of illuminating signal is recognized at a given delay and Doppler shift in the surveillance signal. From the position of the correlation peak in the CAF both the bistatic range and bistatic velocity of a target can be estimated.

The prior information about the position or velocity of a target, from some external source, can be used to limit the range of arguments in the CAF (4) and to reduce the computational effort needed to determine precise values of the estimated parameters. For example, in the case of the radar application for the tracking of satellites, known information about their trajectories and velocities can be additionally employed.

The calculation of the CAF as shown above is equivalent to assuming a linear model of movement with moderate velocity in the bistatic coordinate system. Then, we can assume that the Doppler velocity is constant and the range does not change more than one range cell. Such a simplified assumption is only valid for a sufficiently short integration time. In the experiments presented in the paper, the integration time was equal to 0.5 s which justifies this assumption.

As in the case of the adaptive filtering, several methods can be employed for computationally effective calculation of the CAF. The most common methods known from the literature are the Fourier transform of lag product method and the filter bank method. In our implementation, the Fourier transform of lag product method described in [5] (pp. 135–136) was used.

The automatic detection of peaks in the CAF was performed with the technique known as Constant False Alarm Rate (CFAR), which is based on the thresholding with a threshold calculated from average of data values in the vicinity of the cell under test. In the case of passive radars, the vicinity is defined both in the range and Doppler dimension of the CAF. After thresholding, the exact peak location in both dimensions is estimated to form the detection report containing the bistatic range and the bistatic velocity of a target. In our experiment, we simply find the center of mass of the thresholded data, using the absolute values of the CAF as weights.

Further processing steps in the complete passive radar would include: fusing the detection reports from several transmitter-receiver pairs, finding the unambiguous location of targets, tracking targets usually with some form of Kalman filter. As our experiment was aimed only at presenting a proof of concept related to the detectability of targets using a LOFAR station, these steps were not implemented at the moment.

## 3. Results of Experiments

The simplified experiments were conducted to verify the ability of the detection of targets by the passive radiolocation system using the LOFAR PL610 station, located in Borowiec near Poznan, as the receiver. The closest DAB+ transmitters were selected in order to use the LOFAR station in Borowiec for receiving both the reference and surveillance signals reflected from airplanes. During both experiments that were held in April and December 2019, two tiles out of all 96 HBAs were steered at the digital radio transmitters in Srem and Piatkowo (compare to Figure 7), and the rest of them was directed towards the surveillance space—the air corridor above Swarzedz, where planes often occur. The TBB mode of signal acquisition, discussed in Section 2.3, was used, so that the signals from the tiles could be recorded for about 1 s with 200 MHz sampling frequency. The data was next processed with use of Python and MATLAB.

To verify the results of experiments, in particular the estimated parameters of targets, a dedicated application was prepared. It makes use of the data from flightradar24 [39], which includes flight tracking information. The dedicated application calculates the airplane bistatic parameters, i.e., the bistatic range and bistatic velocity for a given illuminator, from the flightradar24 data.

**Figure 9:**
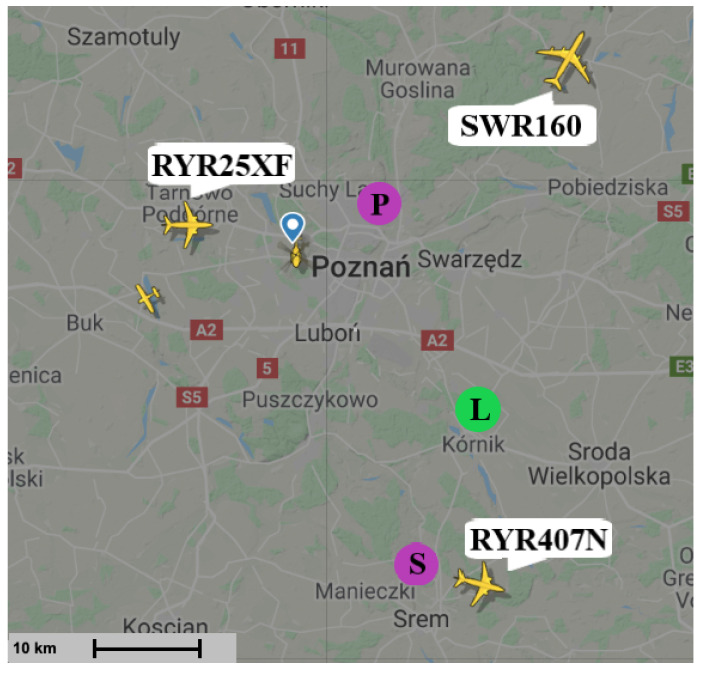
Situation in the air during measurement—April 2019 [39].

**Figure 10:**
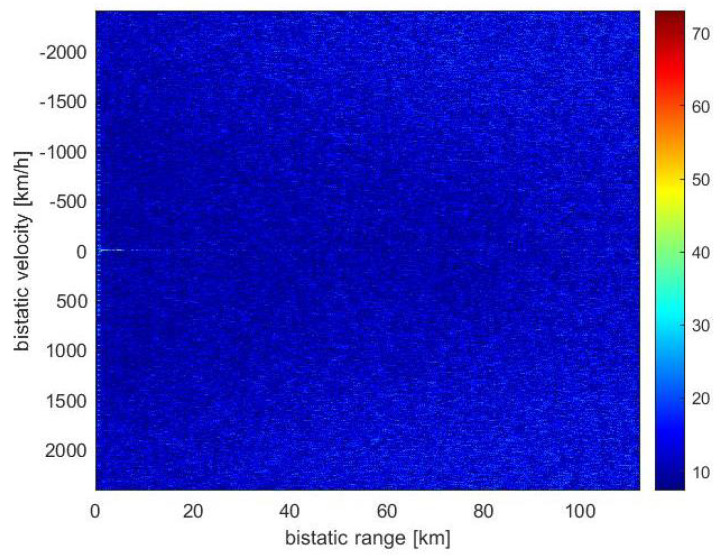
Range-velocity map without applying adaptive filters.

**Figure 11:**
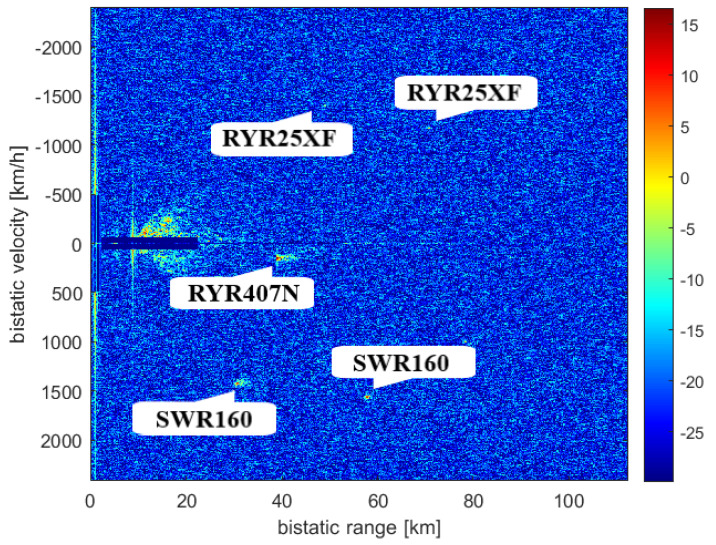
Range-velocity map after applying adaptive filters.

The targets that could be detected at the time of the measurement (14 April 2019) are seen in Figure 9. The results of the CAF computation using the reference signal from Srem transmitter and the surveillance signal are presented in Figure 10. Without applying the adaptive filtering for the removal of the direct-path signal and clutter, no target could be detected. Figure 11 shows the CAF after adaptive filtering. The results emphasize the relevance of this stage of processing, since removing the direct-path transmitter signal and clutter allows detecting the presented targets. It is worth noticing that the total power of the surveillance signal was reduced using the adaptive clutter canceller by about 40 dB. The reduction of the direct-path signal leakage in the CAF, expressed as a difference of the CAF value for the zero bistatic velocity and zero bistatic range cell, before and after the adaptive filtering, reached almost 85 dB.

Due to the fact that both transmitters operate in a single frequency network, the measurement includes echoes for which the reference signal was from the illuminator in Piatkowo as well. It is generally possible to use multiple SFN emitters as the sources of reference signals; however, due to the greater power of Srem station we decided to ignore Piatkowo reference signal this time. The reference signal was received using the LOFAR tile no. 0 and the echo signal using the tile no. 8.

**Figure 12:**
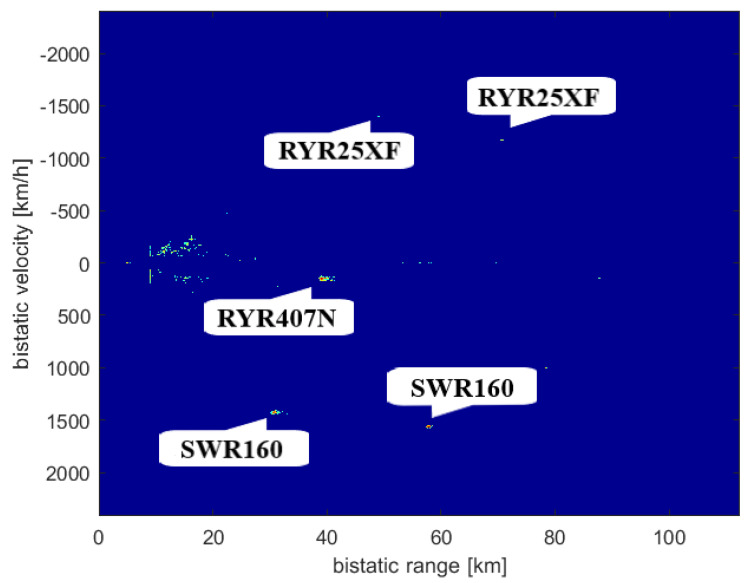
Range-velocity map after CFAR.

The results of the CFAR algorithm processing, with the threshold equal to 12 dB, are presented in Figure 12. The CFAR algorithm compares the level of each cell to the averaged level of its neighbourhood, which is assumed to be noise, so that it could be decided whether that cell contains a target echo or not. Figure 12 shows the detections of three objects: RYR25XF, SWR160, and RYR407N. Both the peaks related to the SWR160 and RYR25XF planes occurred twice as the result of the reflection of signals from both Srem and Piatkowo transmitters. The bistatic range and bistatic velocity of the airplanes for two illuminators are presented in Table 1 (transmitter in Srem, S in Figure 9) and Table 2 (transmitter in Piatkowo, P in Figure 9). Both tables show the comparison of the bistatic parameters estimated using the results of passive signal processing and the results calculated on the basis of the corresponding flightradar24 data. Because of different distances between LOFAR and transmitters (the signal from Piatkowo transmitter is 15.5 s delayed comparing to the signal from Srem transmitter), the estimated parameters required the appropriate correction, which was taken into account in Table 2. The echoes from the RYR25XF plane, calculated using the data from flightradar24, should have appeared at the bistatic distances equal to 70.54 km (Srem) and 45.10 km (Piatkowo) and this target was detected by our passive radiolocation system at 70.65 km and 44.19 km, respectively. For the bistatic velocity of the RYR25XF plane, the results were −1173 km/h (on the basis of the flightradar data) and −1177 km/h (from passive radiolocation system) for the transmitter in Srem, and −1394 km/h and −1403 km/h for the transmitter in Piatkowo. The results obtained for all planes are in Table 1 and Table 2. The RYR407N plane was detected only for the illuminator in Piatkowo because of its position. Generally speaking, the bitatic parameters estimated by means of passive radiolocation are close to the bistatic parameters calculated on the basis of the flightradar24 data. Some discussion of the reasons for the differences is in the next section.

As was already mentioned, in the passive radiolocation, a target might be unambiguously located at the intersection of at least three ellipsoids [31,40]. Each ellipsoid corresponds to a pair of the transmitter and receiver. The position of the transmitters, as well as the receivers, should be known with high accuracy, as the position errors influence on the accuracy of target localisation [41]. Taking advantage of the airplane’s altitude obtained from the flightradar24 data allowed us to reduce the problem to a two-dimensional case and estimate both the distance and azimuth, despite only two transmitter-receiver pairs were available. In this case, our goal is to find the intersection of two ellipses.

**Figure 13:**
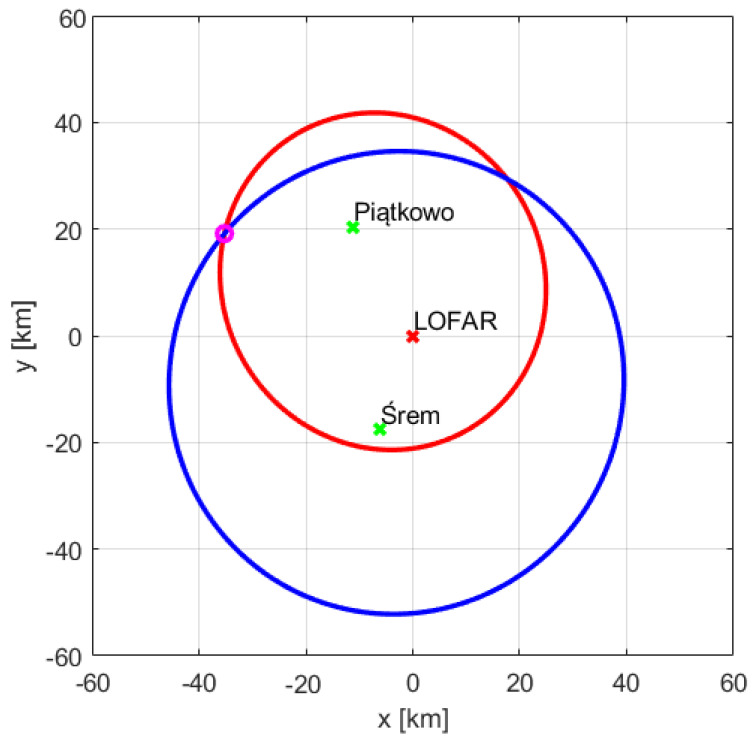
Intersection of ellipses for RYR25XF.

**Figure 14:**
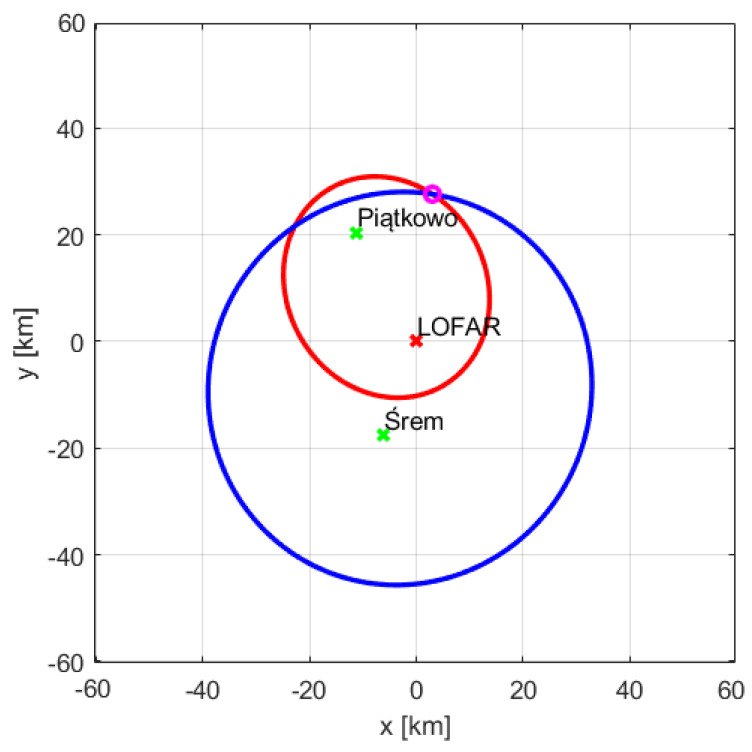
Intersection of ellipses for SWR160.

Figure 13 and Figure 14 show the ellipses obtained for both transmitter-receiver pairs, i.e., Srem-LOFAR and Piatkowo-LOFAR. The ellipses intersections correspond to the positions of the RYR25XF and SWR160 planes, respectively. The LOFAR station (receiver) is in the center of the coordinate system both in Figure 13 and Figure 14, and the foci of the ellipses are in the places where there are the transmitters and the LOFAR station. The ellipses intersects in two points, so the target location requires choosing more probable intersection (Table 3). When determining the target parameters, the height of the transmitter antenna centers was also taken into account, in Srem—250 m above the sea level, and in Piatkowo—128 m above the sea level. The described method of a target location could be applied only to those objects that were detected twice, and in our case, the measurement had to contain reflections whose sources were all available transmitters (Table 1 and Table 2). The RYR25XF plane, according to the flightradar data, was 39.94 km away with the azimuth of 297° to the LOFAR station, the intersection of the ellipses obtained by radar signal processing was at the distance 39.76 km and the azimuth of 298°. The second plane for which the echoes from two transmitters were found—SWR160, was supposed, according to flightradar24 data, to be at the distance of 27.32 km and 10° azimuth. As a result of intersecting the ellipses, the distance of 27.48 km and the azimuth equal to 10° were estimated.

**Figure 15:**
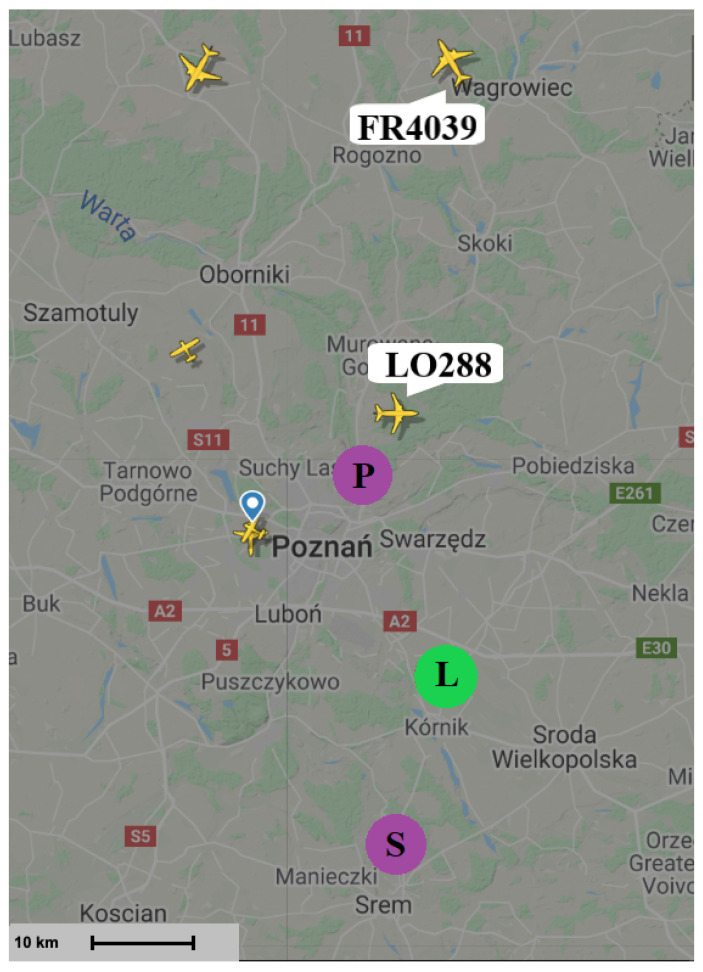
Situation in the air during measurement—December 2019 [39].

**Figure 16:**
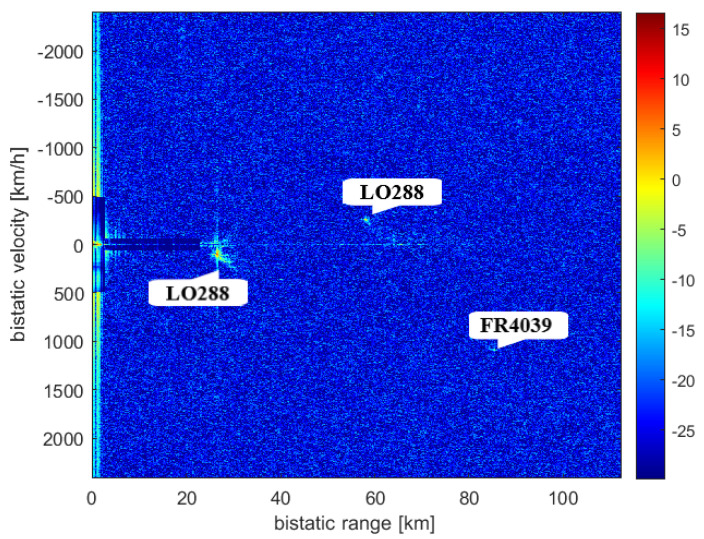
Range-velocity map.

**Figure 17:**
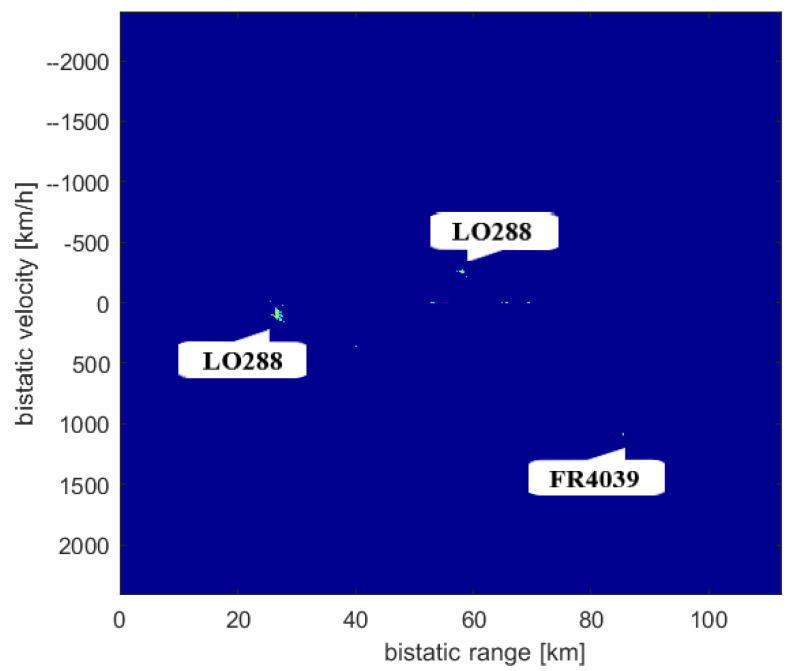
Range-velocity map after CFAR.

The second experiment whose results are presented in this paper took place on 10 December 2019, and the situation in the air near the LOFAR station in Borowiec at the time of measurements is shown in Figure 15. The technique of directing tiles in two directions: towards the transmitter in Srem and towards the air corridor above Swarzędz, was repeated. The recorded data were processed in the same way as the data from April 2019 and the CAF obtained in this case are presented in Figure 16 and Figure 17. Despite having been a few objects in the air, only two of them were detected: the LO288 plane echoes twice, as the result of reflection of signal emitted by both transmitters in Srem (Table 4) and Piatkowo (Table 5). Even though the threshold of CFAR algorithm was only 12 dB, it was impossible to detect the second echo of the FR4039 plane.

**Figure 18:**
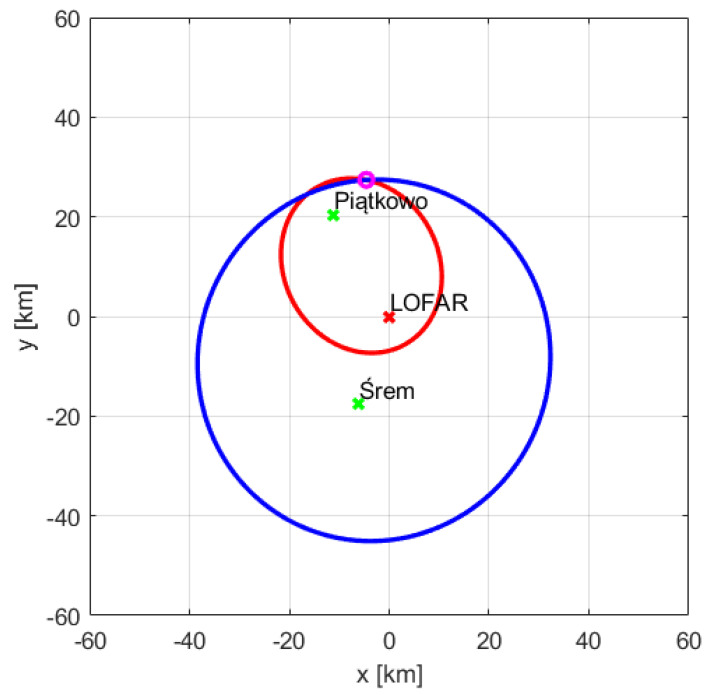
Intersection of ellipses for LO288.

The LO288 plane was detected using the reference signal transmitted from Srem at the bistatic distance of 58.19 km and for the speed of −254 km/h. The bistatic range and velocity calculated on the basis of the flightradar24 data were 57.93 km and −249 km/h, respectively. The echo of this object was also detected for the signal from the transmitter in Piatkowo. The bistatic distance of 21.83 km and the bistatic velocity of 113 km/h were expected on the basis of the flightradar24 data, while the results of the parameters estimation resulted in 21.76 km and 100 km/h, respectively. Using two estimations of the LO288 plane parameters the appropriate ellipses were created and shown in Figure 18. Consequently, the range towards LOFAR and azimuth could be calculated and the results are presented in Table 6. The distance of the LO288 plane from the LOFAR station in Borowiec was determined, which is 30.41 km and the azimuth angle in relation to it is 348°—these values match almost exactly the distance and azimuth calculated on the basis of the data from the flightradar24.

Due to the short recording time, we were not able to estimate statistical measurement errors. In the experiments, the range cell was equal to 150 m and the velocity cell was equal 2.68 m/s (9.65 km/h). The majority of the differences between the PCL-based estimates and the flightradar24-based estimates, shown in Table 1, Table 2, Table 4 and Table 5, are less or comparable to the size of the corresponding cells. However, from our experience in passive radars, both the bistatic range and bistatic velocity errors can reach the level of 0.1–0.5 of the size of a resolution cell. The errors are inversely proportional to the root of the SNR after coherent integration [5]. So after application of more sophisticated methods for signal processing and parameter estimation, we can expect the range errors of 15–75 m and the velocity errors of 0.3–1.2 m/s (1–5 km/h).

Another attempt was made to determine the speed of objects in the Cartesian system, based on the position of the objects and their bistatic velocities. However, to obtain correct results, signals from at least three transmitter-receiver pairs are needed, and this issue was postponed until further field trials.

## 4. Discussion and Conclusions

The analytical studies and the results of the conducted experiment show that a LOFAR station can work as the receiver in a passive radiolocation system. The analyses presented in the first part of the paper, in Section 2.2, show that theoretical detection range is sufficient for the detection of satellites and it will be possible to detect and track space objects using a LOFAR station as the receiver and a set of distant transmitters as illuminators of opportunity. The experimental results presented in Section 3, where aerial targets were detected using the close DAB+ transmitters, constituted a first proof of concept of the LOFAR usage for the passive radiolocation and encouraged taking next steps towards a LOFAR-based satellite tracking system. The obtained results, achieved by using only one tile of the LOFAR antenna, shows that with 0.5 s integration time it is possible to detect aerial targets at the distance about 80 km. The use of full antenna with circa about 20 dB greater gain than a single tile, and the longer integration time, for example adding more than 20 dB gain, will enable us to detect targets at more than 10 times longer distances, proving the theoretical analyses of the space target detection. An additional gain could be achieved by using several illuminators of opportunity and several LOFAR receiving stations.

The obtained results of the estimation of the target bistatic parameters as well as in Cartesian coordinates differ slightly for some targets from parameters calculated on the basis of the flightradar24 data. The differences result from simplifying assumptions taken into account in the processing of echo signals as well as from calculation of the reference parameters based on data from the flightradar24. The files with the flightradar24 data for particular planes are provided at relatively long intervals and the flight parameters had to be interpolated at the given moment when the measurement by means of LOFAR station was performed. On the other hand, the accuracy of the transponders which send the data about flights is limited and the data may contain some inaccuracies. Another reason of the occurring differences is related to the use of only two transmitter-receiver pairs instead of at least three pairs, and taking certain assumptions allowing determining approximate values of the target distance and azimuth. Although only two illuminators broadcasting in the radio-telescope frequency range were found near the LOFAR station, the obtained estimations of target parameters are in general satisfactory.

Since the presented concept was initially proved, the research on other possibilities related to the use of the LOFAR infrastructure in the passive radiolocation will be continued. Firstly, the possibilities of processing of LOFAR narrowband data recorded in the standard mode of LOFAR station operation, which is usually used by astronomers, should be investigated. Using this mode, we will be able to collect significantly longer signals, to use tracking and fusing techniques, and to perform statistical analyses. This mode seems to be a good basis for the development of the prototype of a real-time passive radiolocation system, which can be built as a result of some additions to the hardware of the LOFAR station and the development of the appropriate software for the end-to-end signal processing. In this mode, the LOFAR station provides digital signals in narrow 195.3125 kHz subbands, which should be combined to reconstruct the frequency bands of the signals transmitted by the illuminators of interest. Different frequency ranges are available in that mode, which is why those recordings may allow to make use of a greater number of illuminators. Another very important research area is related to beamforming for passive radar purposes. Some preliminary results on investigations on this subject were presented in [9].

Further steps may include experiments with recordings derived from more LOFAR stations, so that a greater detection range could be achieved. Moreover, the possibility of using the LOFAR station at the same time for both radio astronomy and radiolocation purposes is going to be verified. The biggest challenge is to exploit transmitters that are not local to the LOFAR station bu at the distance of a few thousands of kilometres from LOFAR receiver. To achieve such goal the dedicated reference signal receivers have to be designed and synchronised with the LOFAR network. This is necessary because the distant transmitter direct signals cannot be received within the receiving LOFAR station (as it was done in the described experiment). The experiments in this area are scheduled for 2021.

Also, the use of a long integration time with an advanced motion compensation for orbital targets and the multistatic approach, where several LOFAR receivers and several transmitters of opportunity are exploited, are the necessary techniques to be mastered in the process of designing and constructing the system which may be expected to provide a low cost space surveillance above Europe. As these techniques are already in use, for example for sea targets [42], the space surveillance system using the presented concept may be considered feasible in the light of the results presented.

## Figures and Tables

**Table 1:** Bistatic target parameters for the transmitter in Srem—April 2019.

Object	Flightradar Bistatic Range [km]	Estimated Bistatic Range [km]	Flightradar Bistatic Velocity [km/h]	Estimated Bistatic Velocity [km/h]
RYR25XF	70.54	70.65	−1173	−1177
SWR160	57.60	57.79	1568	1561

**Table 2:** Bistatic target parameters for the transmitter in Piatkowo—April 2019.

Object	Flightradar Bistatic Range [km]	Estimated Bistatic Range [km]	Flightradar Bistatic Velocity [km/h]	Estimated Bistatic Velocity [km/h]
RYR25XF	45.10	44.19	−1394	−1403
RYR407N	34.63	34.60	138	147
SWR160	25.96	26.12	1425	1423

**Table 3:** Target range and azimuth—April 2019.

Object	Flightradar LOFAR–Target Range [km]	Estimated LOFAR–Target Range [km]	Flightradar Azimuth [°]	Estimated Azimuth [°]
RYR25XF	39.94	39.76	297	298
SWR160	27.32	27.48	10	10

**Table 4:** Bistatic target parameters for the transmitter in Srem—December 2019.

Object	Flightradar Bistatic Range [km]	Estimated Bistatic Range [km]	Flightradar Bistatic Velocity [km/h]	Estimated Bistatic Velocity [km/h]
LO288	57.93	58.19	−249	−254

**Table 5:** Bistatic target parameters for the transmitter in Piatkowo—December 2019.

Object	Flightradar Bistatic Range [km]	Estimated Bistatic Range [km]	Flightradar Bistatic Velocity [km/h]	Estimated Bistatic Velocity [km/h]
LO288	21.83	21.76	113	100
FR4039	80.78	80.77	1078	1086

**Table 6:** Target range and azimuth—December 2019.

Object	Flightradar LOFAR–Target Range [km]	Estimated LOFAR–Target Range [km]	Flightradar Azimuth [°]	Estimated Azimuth [°]
LO288	30.40	30.41	348	348

## Data Availability

Data available on request.

## Abbreviations

The following abbreviations are used in this manuscript:

ASTRON	the Netherlands Institute for Radio Astronomy
BLF	Block Lattice Filter
CAF	The Cross-Ambiguity Function
CFAR	Constant False Alarm Rate
DAB	Digital Audio Broadcasting
DVB-T	Digital Video Broadcasting—Terrestrial
EIRP	Effective Isotropical Radiated Power
HBA	the High Band Antennas
ILT	International Lofar Telescope
LBA	the Low Band Antennas
LMS	Least Mean Square
LOFAR	The Low-Frequency Array
LSL	Least Squares Lattice
PCL	Passive coherent location
RLS	Recursive Least Squares
SFN	Single Frequency Network
SNR	Signal-to-Noise Ratio
TBB	Transient Buffer Board
